# Multicenter study of risk factors of unplanned 30‐day readmissions in pediatric oncology

**DOI:** 10.1002/cnr2.1343

**Published:** 2021-02-02

**Authors:** Kamila Hoenk, Lilibeth Torno, William Feaster, Sharief Taraman, Anthony Chang, Michael Weiss, Karen Pugh, Brittney Anderson, Louis Ehwerhemuepha

**Affiliations:** ^1^ Children's Hospital of Orange County Orange California USA; ^2^ Beckman Research Institute City of Hope Duarte California USA; ^3^ Schmid College of Science and Technology Chapman University Orange California USA

**Keywords:** neoplasms, oncology, pediatrics, unplanned readmission

## Abstract

**Background:**

Pediatric oncology patients have high rates of hospital readmission but there is a dearth of research into risk factors for unplanned 30‐day readmissions among this high‐risk population.

**Aim:**

In this study, we built a statistical model to provide insight into risk factors of unplanned readmissions in this pediatric oncology.

**Methods:**

We retrieved 32 667 encounters from 10 418 pediatric patients with a neoplastic condition from 16 hospitals in the Cerner Health Facts Database and built a mixed‐effects model with patients nested within hospitals for inference on 75% of the data and reserved the remaining as an independent test dataset.

**Results:**

The mixed‐effects model indicated that patients with acute lymphoid leukemia (in relapse), neuroblastoma, rhabdomyosarcoma, or bone/cartilage cancer have increased odds of readmission. The number of cancer medications taken by the patient and the administration of chemotherapy were associated with increased odds of readmission for all cancer types. Wilms Tumor had a significant interaction with administration of chemotherapy, indicating that the risk due to chemotherapy is exacerbated in patients with Wilms Tumor. A second two‐way interaction between recent history of chemotherapy treatment and infections was associated with increased odds of readmission. The area under the receiver operator characteristic curve (and corresponding 95% confidence interval) of the mixed‐effects model was 0.714 (0.702, 0.725) on the independent test dataset.

**Conclusion:**

Readmission risk in oncology is modified by the specific type of cancer, current and past administration of chemotherapy, and increased health care utilization. Oncology‐specific models can provide decision support where model built on other or mixed population has failed.

## 1 INTRODUCTION

In 2020, there were 1.7 million new cases of cancer and 0.6 million projected cancer deaths in the United States.^1^ It is estimated that more than 15 000 new pediatric cases of cancer are diagnosed yearly in the United States, which will result in more than 1900 cancer‐related deaths.^2^ Furthermore, 1 in 285 children are diagnosed with cancer before the age of 20 years, indicating that many hospitals across the country will be treating children with cancer.^2^ Recent improvements in cancer therapies and treatments have resulted in an increase in the 5‐year net survival rate of children with cancer, which is estimated to be approximately 80% in high‐income countries.^3^ Treatment of cancer in children often requires multiple hospitalizations including planned encounters for chemotherapy, immunotherapy, or radiotherapy. Unplanned readmissions among these patients are usually undesirable and expensive. In the adult population, readmissions among oncology patients were associated with a median cost of $9220 in 2010 and a total of $9.3 million between 1992 and 2010 (SEER‐Medicare linked database).^4^, ^5^ Patients diagnosed with neoplastic conditions are most at risk for unplanned readmissions. This risk is increased by administration of chemotherapy, immunotherapy, or radiation therapy.^6^, ^7^, ^8^, ^9^, ^10^ There is a paucity of research into risk factors among pediatric oncology patients in comparison to general pediatric or adult models, due to the relative rarity of pediatric cancers. Research development in this field of pediatric oncology is further hampered by the need for valid pediatric oncology‐specific models that estimate the true risk of hospital readmission within this group of high‐risk patients.

In this study, we applied advanced statistical methods to determine cancer‐specific risk factors of readmission in pediatric oncology using multicenter data on patients diagnosed with cancer. We hypothesize that the risk for hospital readmission is dependent on the type of cancer, administration of chemotherapy, the number of cancer medications, and putative risk factors of readmission. The development of oncology‐specific models for readmission is, therefore, important for risk stratification among patients with cancer, given that there is an elevated baseline risk for readmission in oncology, and that general readmission models fail in this high‐risk population. We developed a mixed‐effects (statistical) model as our model for inference. Data from the Cerner Health Facts Database (referred to as Health Facts DB from here on) were utilized to train and test the oncology readmission models.

## 2 METHODS

This study was approved by the Children's Hospital of Orange County Institutional Review Board (IRB 180857) and uses the Health Facts DB (now referred to as the Cerner Real World Data^11^). The Health Facts DB consists of data captured by Cerner Corporation from over 100 US healthcare systems and over 650 facilities (in 2018) that are aggregated and organized into consumable datasets to facilitate research and reporting. The data were encrypted and secured to maintain patient confidentiality and ensure compliance with HIPAA privacy regulations. The Health Facts DB consists of clinical database tables with data on patient demographics, encounters, medications, laboratory tests, clinical events, and diagnoses among others. At the time of this study, the database consisted of 6.9 million patients and 503.8 million encounters across all care settings. The model developed in this study was built using a subset of data from the database based on a priori inclusion criteria.

Data from patients less than 21 years that were hospitalized with a neoplastic condition between 2000 and 2017 were retrieved from the database. Inclusion criteria for hospitals in this study were that they (a) contributed data to all the database tables required for this study and (b) had data from at least 500 pediatric admissions for neoplastic conditions within the time period of the study (from 2000 to 2017). Planned encounters were identified using the ICD‐10‐CM diagnosis codes, Z00‐Z13, which indicate visits for examinations and screenings as well as chemotherapy, immunotherapy, and radiation therapy; and encounters for specific health care such as surgeries (Z40‐Z53).^12^ The 30‐day readmission status was calculated and all planned readmissions, defined by the conditions previously indicated, were excluded.

Nine of the most common childhood cancers,^1^ consisting of acute lymphoid leukemia (ALL), acute myeloid leukemia (AML), brain cancer, neuroblastoma, Wilms tumor, Hodgkin's Lymphoma (HL), non‐Hodgkin's Lymphoma (NHL), rhabdomyosarcoma, and cancers of the bone and cartilage were selected, with all other rarer cancers grouped together as “other cancers.”

Several categories of data were retrieved as covariates, including demographics and payer, healthcare resource utilizations, neoplastic conditions, hematological conditions, other comorbidities, and the number of cancer medications administered. A comprehensive list of approved pediatric cancer medications was retrieved from Barone et al and used to determine the number of unique cancer medications administered during each visit.^13^ A look‐back period of 6 months from the index/current visit was used to capture data on prior healthcare resource utilization (such as previous visits, previous readmissions). All ICD‐9‐CM codes were converted to ICD‐10‐CM for identification of classes of diseases/conditions. We chose classes of diseases according to the chapters of ICD‐10‐CM codes such as conditions affecting digestive (K00‐K95) and genitourinary (N00‐N99) systems—all non‐neoplastic conditions/comorbidities considered are listed in Table 1.

**TABLE 1:** Summary statistics

Variable	Levels	Not readmitted	Readmitted
		*n (%) for categorical data or mean (SD) for continuous*	
Age (y)	‐	9.16 (5.72)	8.96 (5.85)
Sex	Female	6374 (44.22)	4675 (45.69)
	Male	8039 (55.78)	5558 (54.31)
Race/Ethnicity	Caucasian	8518 (59.10)	6031 (58.94)
	Hispanic	420 (2.91)	338 (3.30)
	African American/Black	1774 (12.31)	1380 (13.49)
	Asian/Pacific Islander	210 (1.46)	201 (1.96)
	Native American	68 (0.47)	46 (0.45)
	Others/Unknown	3423 (23.75)	2237 (21.86)
Payer	Commercial	5550 (38.51)	4022 (39.30)
	Governmental	6124 (42.49)	4392 (42.92)
	Self‐pay	186 (1.29)	132 (1.29)
	Others	2553 (17.71)	1687 (16.49)
**Most common pediatric cancers**			
Acute lymphoid leukemia (ALL)	No	9591 (66.54)	7561 (73.89)
	Yes, not in remission	2692 (18.68)	1466 (14.33)
	Yes, in remission	1830 (12.70)	872 (8.52)
	Yes, in relapse	300 (2.08)	334 (3.26)
Acute myeloid leukemia (AML)	No	13 736 (95.30)	9723 (95.02)
	Yes, not in remission	342 (2.37)	279 (2.73)
	Yes, in remission	225 (1.56)	152 (1.49)
	Yes, in relapse	110 (0.76)	79 (0.77)
Brain cancer	No	12 067 (83.72)	9117 (89.09)
	Yes	2346 (16.28)	1116 (10.91)
Neuroblastoma	No	13 541 (93.95)	9108 (89.01)
	Yes	872 (6.05)	1125 (10.99)
Wilms tumor	No	13 849 (96.09)	9779 (95.56)
	Yes	564 (3.91)	454 (4.44)
Hodgkin's lymphoma	No	13 937 (96.70)	9924 (96.98)
	Yes	476 (3.30)	309 (3.02)
Non‐Hodgkin's lymphoma	No	13 646 (94.68)	9631 (94.12)
	Yes	767 (5.32)	602 (5.88)
Rhabdomyosarcoma	No	13 610 (94.43)	9431 (92.16)
	Yes	803 (5.57)	802 (7.84)
Bone/cartilage cancer	No	13 365 (92.73)	8760 (85.61)
	Yes	1048 (7.27)	1473 (14.39)
Other cancers	No	11 983 (83.14)	8712 (85.14)
	Yes	2430 (16.86)	1521 (14.86)
**Cancer‐related therapies/treatment**			
Chemotherapy	No	10 100 (70.08)	5557 (54.30)
	Yes	4313 (29.92)	4676 (45.70)
Bone marrow transplant	No	13 867 (96.21)	9904 (96.78)
	Yes	546 (3.79)	329 (3.22)
Number of cancer medications	‐	0.92 (1.18)	1.09 (1.31)
Number of previous visits for chemotherapy (prior 30 d)	‐	0.22 (0.47)	0.36 (0.58)
**Healthcare resource utilization variables**			
Length of stay	0, 1	2645 (18.35)	1451 (14.18)
	2, 3	4592 (31.86)	3183 (31.11)
	4, 5, 6	3499 (24.28)	3146 (30.74)
	7 or more	3677 (25.51)	2453 (23.97)
Emergent admission	No	5574 (38.67)	5177 (50.59)
	Yes	8839 (61.33)	5056 (49.41)
Is index/current visit itself a readmission	No	8378 (58.13)	3392 (33.15)
	Yes, unplanned	3278 (22.74)	3254 (31.80)
	Yes, planned	2757 (19.13)	3587 (35.05)
Previous ED visits (prior 6 mo)	‐	0.62 (1.26)	0.67 (1.13)
Number of previous visits without chemotherapy (prior 6 mo)	‐	1.31 (1.74)	2.29 (2.48)
Previous readmissions (prior 6 mo)	0	9531 (66.13)	4502 (43.99)
	1	2190 (15.19)	1931 (18.87)
	2	1171 (8.12)	1311 (12.81)
	3 or more	1521 (10.55)	2489 (24.32)
**Comorbid conditions**			
Viral or bacterial infections (A00 ‐ B99)	No	11 255 (78.09)	8318 (81.29)
	Yes	3158 (21.91)	1915 (18.71)
Hematological conditions (D50‐D69, D71‐D79)		10 253 (71.14)	7353 (71.86)
		4160 (28.86)	2880 (28.14)
Neutropenia (D70)	No	11 049 (76.66)	8335 (81.45)
	Yes	3364 (23.34)	1898 (18.55)
Immunological conditions (D80‐D89)	No	13 672 (94.86)	9820 (95.96)
	Yes	741 (5.14)	413 (4.04)
Endocrine, nutritional, and metabolic diseases (E00‐E89)	No	10 679 (74.09)	7606 (74.33)
	Yes	3734 (25.91)	2627 (25.67)
Mental, behavioral, and neurodevelopmental disorders (F01‐F99)	No	12 571 (87.22)	9021 (88.16)
	Yes	1842 (12.78)	1212 (11.84)
Nervous system (G00‐G99)	No	11 067 (76.78)	8320 (81.31)
	Yes	3346 (23.22)	1913 (18.69)
Eye, adnexa, ear, mastoid (H00‐H95)	No	12 762 (88.55)	9271 (90.60)
	Yes	1651 (11.45)	962 (9.40)
Circulatory (I00‐I99)	No	12 137 (84.21)	8673 (84.76)
	Yes	2276 (15.79)	1560 (15.24)
Respiratory (J00‐J99)	No	11 414 (79.19)	8579 (83.84)
	Yes	2999 (20.81)	1654 (16.16)
Digestive (K00‐K95)	No	10 572 (73.35)	7481 (73.11)
	Yes	3841 (26.65)	2752 (26.89)
Skin/subcutaneous tissue (L00‐L99)	No	12 684 (88.00)	9017 (88.12)
	Yes	1729 (12.00)	1216 (11.88)
Musculoskeletal and connective tissue (M00‐M99)	No	12 881 (89.37)	9265 (90.54)
	Yes	1532 (10.63)	968 (9.46)
Genitourinary (N00‐N99)	No	11 988 (83.17)	8470 (82.77)
	Yes	2425 (16.83)	1763 (17.23)
Congenital/Chromosomal (Q00‐Q99)	No	13 385 (92.87)	9768 (95.46)
	Yes	1028 (7.13)	465 (4.54)
Injuries and poisoning (S00‐T88)	No	12 916 (89.61)	9339 (91.26)
	Yes	1497 (10.39)	894 (8.74)

An a priori threshold of 100 responses/encounters was selected to guide against absolute rarity or sparsity of variables/covariates and to reduce the potential for problems (such as inestimable or inflated effect sizes) due to statistical separation during model development.^14^ The level of multicollinearity was assessed by estimating the generalized variance inflation factor (GVIF) across the candidate variables^15^, ^16^, ^17^, ^18^, ^19^ and excluding variables with GVIF greater than 4 as a threshold.^19^ Statistical separation and multicollinearity can lead to unstable models and false discoveries, hence the need to address them before model development. Summary statistics of all variables considered during model development are shown in Table 1.

The data were randomly split into a training set (encounters from 75% of the patients) and a test set (encounters from 25% of the patients). A nested mixed‐effects^20^ logistic regression model with the hospitals modeled as the random intercept and patients nested within hospitals was built. The mixed‐effects modeling necessitated splitting the data by unique patients such that all encounters/hospitalizations from the same patient were captured entirely in either the training or the test set. The choice of a mixed‐effects model was driven by the hierarchical nature of the data, wherein patients within the same hospitals are likely to be more correlated with themselves than with patients from other hospitals, and encounter data from the same patient are more likely to be correlated with themselves than encounter data of other patients within the same hospital.

Model selection began by building a full model (including all variables in Table 1) as well as statistical interactions between the following pairs of variables; between the administration of chemotherapy on the index/current visit and all cancers and comorbidities; between number of previous visits for the administration of chemotherapy within the last 30 days and all cancers and comorbidities; and between the number of unique cancer medications and all cancers and comorbidities. These were selected to assess the hypotheses that the risk of readmission due to a neoplastic condition and comorbidities may differ by administration of chemotherapy during the index/current visit, recent history of chemotherapy, and number of cancer medications administered during the index/current visit. Variable selection involved a backward stepwise procedure (by minimization of the Akaike Information Criteria^14^) starting with the two‐way statistical interactions in the full model that was not significant (at alpha level of 0.05). Once all significant interactions were selected, we continued with backward elimination on all other variables, keeping the main effects terms for neoplastic conditions regardless of statistical significance.^21^, ^22^Model performances were measured using the area under the receiver operator characteristic curve (AUROC) and the area under the precision‐recall curve (AUCPR). Values of model sensitivity, positive predictive value, relative risk, and the number needed to evaluate (NNE) at model specificity of 0.80 were calculated. The NNE is defined in the case of a predictive model as the total number of patients who would be flagged as “at risk” to capture one true positive prediction.

## 3 RESULTS

**FIGURE 1:**
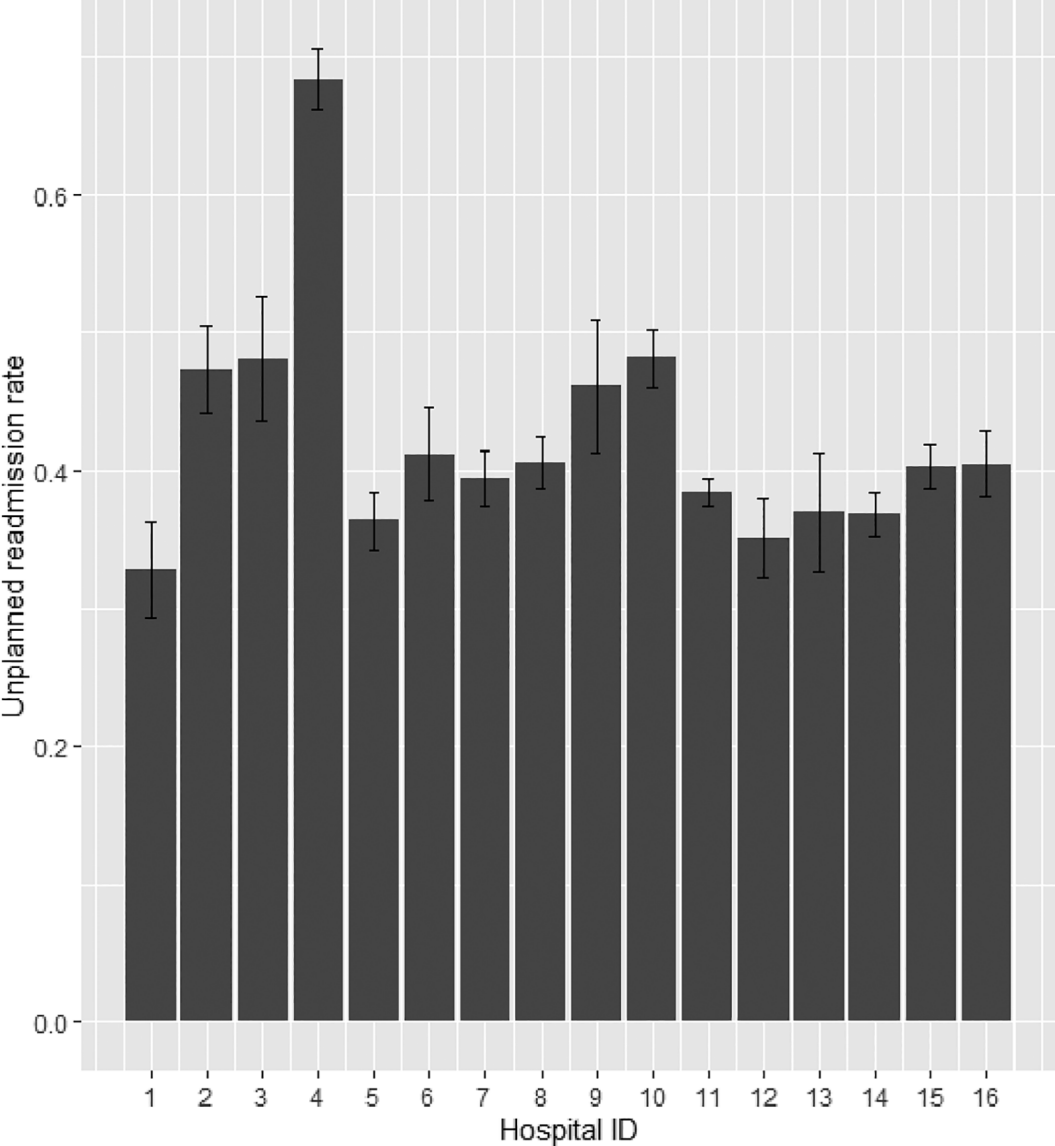
Distribution of unplanned readmission rates across the hospitals

A total of 16 hospitals met the inclusion criteria. There were 10 418 patients and 32 667 corresponding hospitalizations, after excluding planned readmissions. The hospitals contributed an average of 4.3 years of data to this study during the study years (2000‐2017). The average age of the patients was 9 years (SD of 5 years), 44.9% were female, 59.2% were Caucasian, 12.9% African American or Black, 3.1% Hispanic, 1.6% Asian or Pacific Islander, 0.5% Native American, and 22.6% of other or unknown races/ethnicities. The percentage of patients on governmental and commercial insurance was 42.5% and 38.9% respectively, 1.3% were on self‐pay, and the remaining were on another or unknown type of payer source. In terms of length of stay of the index/current visit, 16.6, 31.5, 26.8, and 25.1% of visits were 0‐1, 2‐3, 4‐6, and 7 or more days respectively. The overall 30‐day unplanned readmission rate of the qualifying oncology patients across all institutions was 41.2% for pediatric oncology patients (excluding planned readmissions for chemotherapy treatment). See Figure 1 for the distribution of readmission rates across the 16 hospitals.

After splitting the data into a training and test set, we observed 7814 patients (75%) in the training data and the remaining 2604 patients on the test set. The resulting number of encounters on the training data is 24 646 as shown in Table 1. Summary statistics of the training data on each variable by readmission status are shown in Table 1.

### 3.1 The nested mixed‐effects model

The resulting multivariable nested mixed‐effects logistic regression model included both main effects and two 2‐way statistical interactions as shown in Table 2. Patients most at risk of unplanned readmission (in decreasing order of risk) were patients with Wilms Tumor undergoing chemotherapy (168.0% increase in odds), patients undergoing chemotherapy for neoplasms excluding Wilms Tumor (71.8% increase in odds), patients with bone and cartilage cancer (61.8% increase in odds), patients with neuroblastoma (44.2% increase), patients in relapse for ALL (43.6% increase in odds), and rhabdomyosarcoma (18.2%). There were reduced odds of readmission among patients with brain cancer (11.8% reduction in odds). There were also reduced odds of readmission for patients not in remission for ALL (11.2%) and for patients in remission for ALL (34.4%). In other words, patients in relapse for ALL were identified as the at‐risk group for this cancer. Statistically significant differences were not observed for patients with AML. The number of cancer medications administered was associated with increased odds of readmission. Each unique cancer medication given increases the odds of readmission by 14.6%. The maximum number of unique cancer medications administered to a single patient in this study was 9 with a mean and median of 1 cancer medication per patient in the study. This does not address the difference in risk that may exist by the type of cancer medication administered.

**TABLE 2:** Nested mixed‐effects model

Variables	Levels	Odds ratio	*P* value
Age	‐	0.987 (0.982, 0.993)	<.001
**Cancers and related therapies (no statistical interactions)**			
Acute lymphoid leukemia	No	Reference	<.001
	Yes, not in remission	0.788 (0.721, 0.862)	
	Yes, in remission	0.656 (0.590, 0.729)	
	Yes, in relapse	1.436 (1.201, 1.718)	
Acute myeloid leukemia	No	Reference	
	Yes, not in remission	1.158 (0.968, 1.386)	.109
	Yes, in remission	1.047 (0.832, 1.318)	.692
	Yes, in relapse	1.053 (0.768, 1.444)	.750
Brain cancer	Yes	0.782 (0.711, 0.861)	<.001
Neuroblastoma	Yes	1.442 (1.285, 1.619)	<.001
Rhabdomyosarcoma	Yes	1.182 (1.048, 1.332)	.006
Bone/cartilage cancer	Yes	1.618 (1.455, 1.799)	<.001
Number of cancer medications	Yes	1.146 (1.117, 1.175)	<.001
**Comorbidities**			
Endocrine, nutritional, and metabolic disorders	Yes	1.141 (1.067, 1.221)	.001
Congenital and chromosomal diseases	Yes	0.745 (0.657, 0.844)	<.001
Injuries, burns, and poisoning	Yes	0.838 (0.760, 0.925)	<.001
**Healthcare resource utilization**			
Number of previous visits without chemotherapy (prior 6 mo)	‐	1.077 (1.047, 1.108)	<.001
Length of stay (d)	<2	Reference	<.001
	2, 3	1.292 (1.184, 1.410)	
	4, 5, 6	1.466 (1.340, 1.604)	
	7 or more	1.344 (1.219, 1.481)	
Is current visit itself a readmission	No	Reference	<.001
	Yes, unplanned	1.869 (1.722, 2.028)	
	Yes, planned	1.397 (1.250, 1.560)	
Previous readmissions (prior 6 mo)	0	Reference	<.001
	1	1.227 (1.126, 1.336)	
	2	1.281 (1.145, 1.433)	
	3 or more	1.263 (1.087, 1.468)	

Statistical interactions			
Main effects of interaction terms	Levels	Regression coefficient	*P* value
Wilms Tumor	Yes	−0.100	.278
Chemotherapy during index/current visit	Yes	0.541	<.001
Number of previous visits for chemotherapy (prior 30 days)	‐	−0.087	.014
Viral, bacterial, or parasitic infections	Yes	−0.181	<.001
**Two‐way interaction terms**			
Wilms Tumor and chemotherapy	‐	0.545	<.001
Number of previous visits for chemotherapy and viral, bacterial, or parasitic infections	‐	0.291	< .001

**FIGURE 2:**
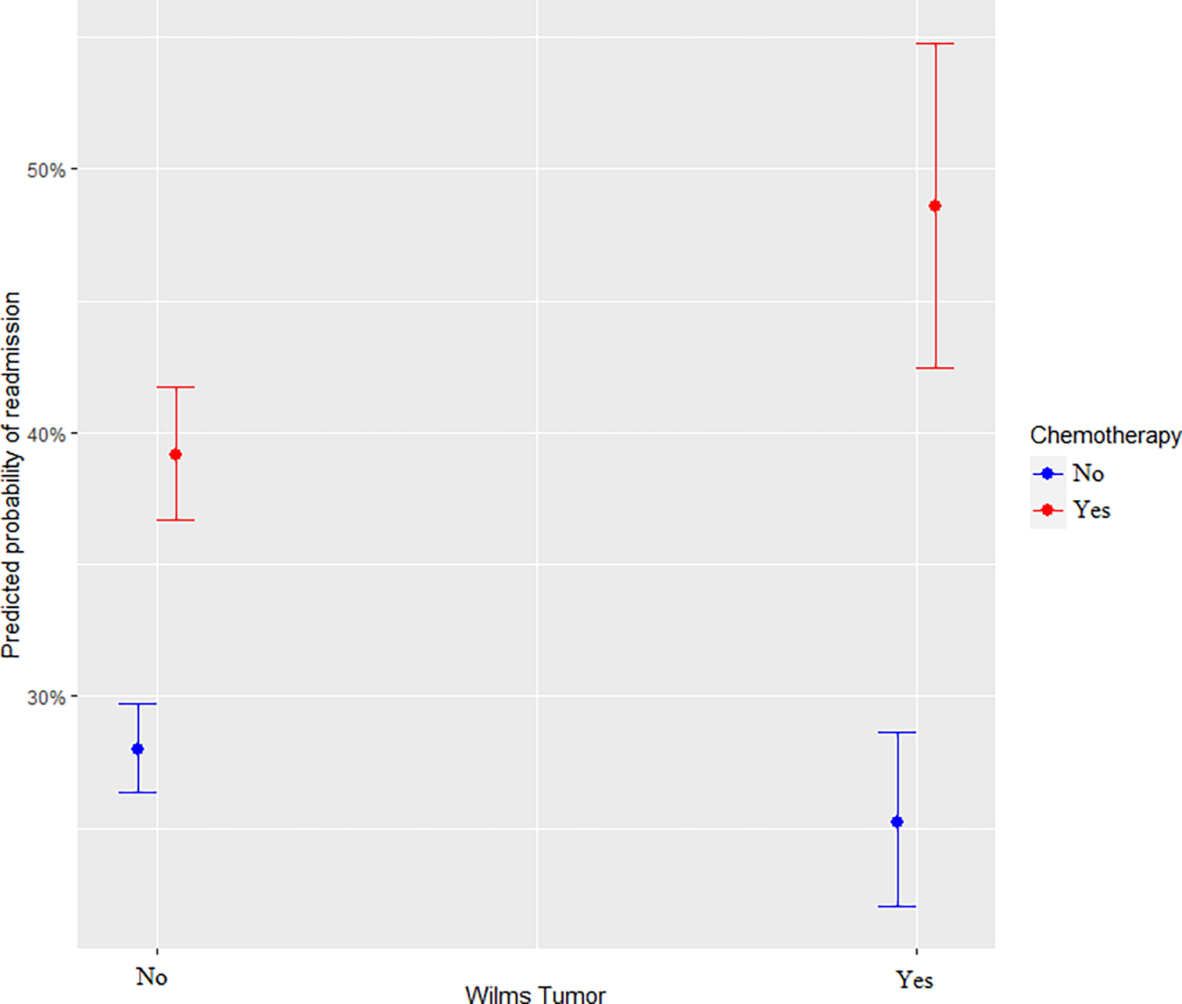
Statistical Interaction between Wilms Tumor and Chemotherapy

**FIGURE 3:**
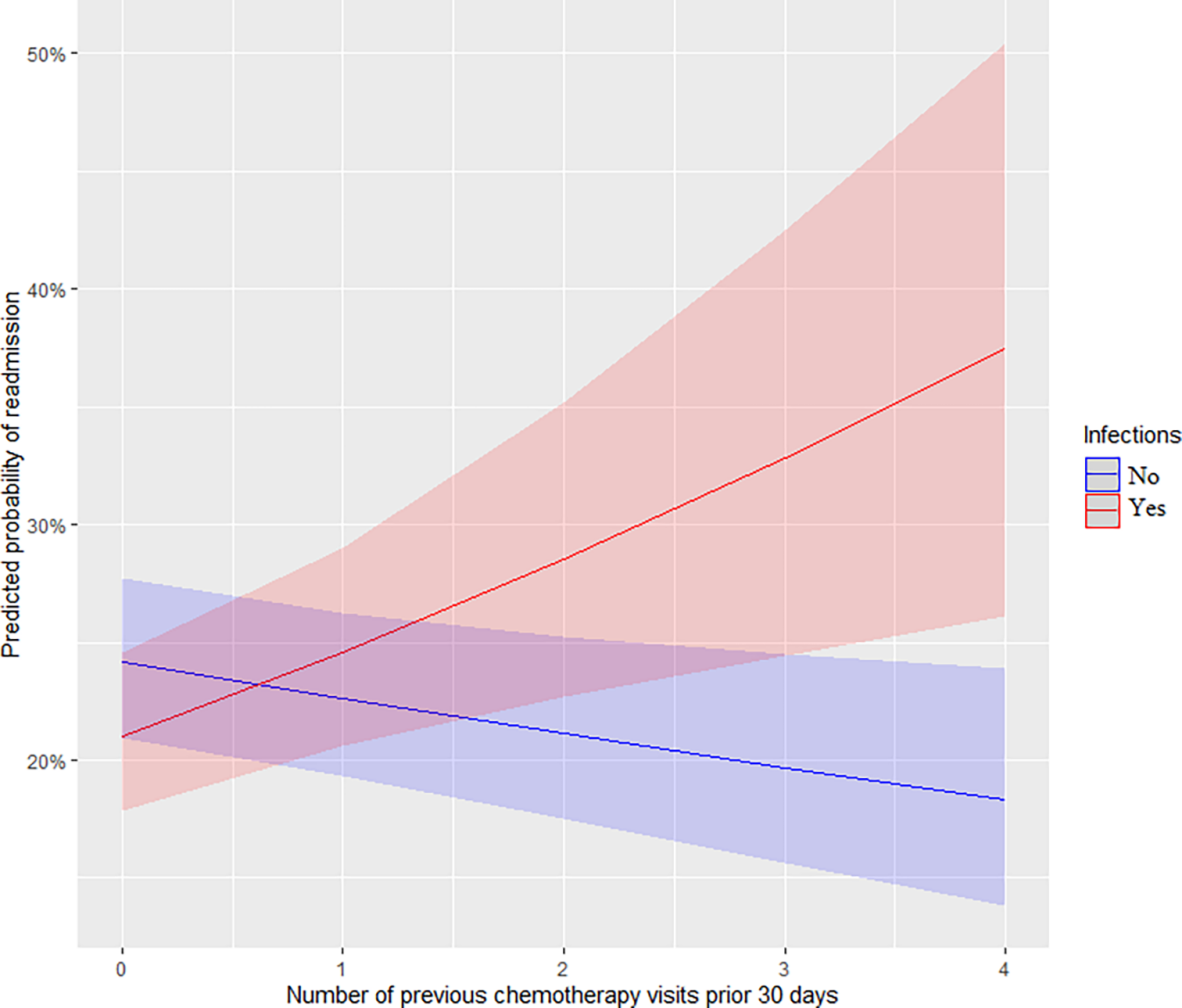
Statistical interaction between the number of visits for chemotherapy within the last 30 days and viral, bacterial, or parasitic infections

The effect of the statistical interaction between Wilms Tumor and the administration of chemotherapy is shown in Figure 2. The interaction indicates that there is a tendency toward lower odds of readmission among patients with Wilms Tumor compared to other cancer types when chemotherapy is not being administered. Administration of chemotherapy increases the risk of readmission regardless of the type of cancer but a significantly higher odds of readmission exist for patients with Wilms Tumor undergoing chemotherapy. The second interaction was between the number of previous chemotherapy visits (within the last 30 days) and infectious diseases (viral, bacterial, and parasitic infections), see Figure 3. The risk of readmission increases with the number of previous chemotherapy visits among patients with infections, but a reversed and less‐steep drop in odds was observed if the patient did not have an infection afterward.

Some putative risk factors of unplanned readmission in the general pediatric population were also found as risk factors within the pediatric oncology patient population.^7^, ^11^ Patients showed a 1.1% decrease in odds of readmission for every age‐positive difference of 1 year. There were 29.2%, 46.6%, and 34.4% increase in odds of readmission for patients with length of stay of 2‐3, 4‐6, and 7 days or more respectively, compared with patients staying 24 hours or less. There were 22.7%, 28.1%, and 26.3% increase in odds of readmission among patients with 1, 2, or 3 more prior readmissions within the 6 months preceding the index/current visit, respectively. There was 86.9% increase in odds for a subsequent readmission if the index/current visit is itself an unplanned readmission from a prior encounter. In contrast, there was a 39.7% increase in odds for readmission if the index/current visit is itself a planned readmission. There was a 7.7% increase in odds for readmission for every previous hospitalization without chemotherapy that occurred during 6 months prior to the index/current encounter.

The nested mixed‐effects model had an area under the receiver operator characteristic curve (AUROC) of 0.714 (0.702, 0.725) on the independent test dataset. At a specificity of 0.80, the sensitivity, positive predictive value, relative risk, NNE, and the predicted probability of readmission threshold (for flagging patients at high risk) are 0.47 (0.46, 0.49), 0.62 (0.60, 0.64), 2.00 (1.90, 2.11), 1.62, and 0.49 respectively. This implies, at a specificity of 0.80, we would expect approximately two true positive predictions from every three patients flagged at high risk by the model and capture 51% of all unplanned readmissions.

The area under the precision‐recall area under the curve was calculated to be 0.614.

## 4 DISCUSSION

Predictive modeling for pediatric oncology hospital readmissions has largely been left unaddressed in medical and data science literature. These patients are, however, universally seen as patients at the highest risk for readmission (as captured in existing models for the general pediatric population).^6^, ^11^, ^12^ The challenge with general pediatric readmissions models is that the models often suffer from poor specificity in stratifying risk in oncology.

Most findings in this study include unmodifiable risk factors, as expected of a comprehensive model built to estimate overall risk. As a result, the development of interventions based on the model cannot be automated given only the information available to and provided from the model. Rather, the overall estimate of risk based on obvious, subtle, modifiable, and unmodifiable risk factors need to be taken into consideration for determining the overall risk. The provider is then presented with the overall risk of readmission as well as both modifiable and unmodifiable risk factors. This information, in addition to the provider's insight and knowledge of the child, should be used in the development of personalized intervention plans. No statistical model on its own suffices in addressing the clinical needs of patients—these models exist to inform, not direct, the provider.

Our results, however, provide a potential opportunity for improvement of the quality of care for pediatric oncology through the identification of pediatric oncology‐specific risk factors. The mixed‐effects model developed had high performance for predicting unplanned readmission such that the mixed‐effects model will capture over 47% of all unplanned readmissions at specificity of 80%. In other words, the mixed‐effects model performance metrics indicate that a significant proportion of patients who will be readmitted will be captured by this model with acceptable levels of false positive predictions.

Based on the mixed‐effects model, the development of intervention protocols around patients who will be predicted at high risk of readmission (in the absence of a future planned encounter within 30 days) may be conceptualized and further refined. Development of effective intervention protocols is nuanced and much more difficult in the presence of unmodifiable risk factors as identified by the nested mixed‐effects model. Previous experience with the adoption of statistical or machine learning models^6^, ^7^ and the development of corresponding intervention protocols indicates that interventions should be tailored to each patient based on the risk factors that contribute to their risk level and following discussions with the patient and family.

The administration of antineoplastic chemotherapy on the index/current visit is associated with the highest risk of readmission regardless of the cancer type. Based on previous studies,^4^, ^6^, ^10^ this result holds true among the pediatric oncology population as well as the general pediatric and adult populations. The strongest risk factor for unplanned readmissions among pediatric oncology patients identified in this study was the administration of chemotherapy for Wilms Tumor. Furthermore, the total number of unique cancer medications administered is associated with increased risk of readmission. This result is averaged over the cancer medications considered, and further studies are required to indicate how the risk of readmission changes due to the specific type of medication administered to treat the cancer.

There are also elevated odds for readmission among patients with ALL in relapse, bone and cartilage cancer, neuroblastoma, and rhabdomyosarcoma. Patients who are in relapse for ALL are often in need of a strong reinduction of chemotherapy.^23^, ^24^ Additionally, these patients may be sick at the time of relapse, and therefore require more clinical attention and resources than newly diagnosed ALL patients. Neuroblastoma and rhabdomyosarcoma also showed an increase in odds of readmission. These cancer types are known to have aggressive metastasizing behaviors.^25^ Additionally, similar to the treatment of higher‐risk leukemia, neuroblastoma is known to be treated with high‐risk transplant procedures in addition to chemotherapy, surgery, and radiation.^25^ Each of these procedure types is associated with immediate and long‐term risk factors for infection, myopathy, and other complications.^23^, ^25^, ^26^ Rhabdomyosarcoma is the third most common extracranial solid tumor in pediatrics and one of the cancers for which prognosis for advanced stages and those harboring deleterious genomic abnormalities has not improved.^27^ Our findings indicate the need for improved quality of care of these patients to reduce unplanned readmission after discharge from the hospital. Brain cancer, conversely, showed a reduction in odds of readmission. This reduction in risk makes clinical sense because brain cancer, when operable, is often treated with surgery and chemotherapy sessions that are not as frequent and intensive.^28^

A common complication from the treatment of cancer is chemotherapy‐induced neutropenia, which has been reported as a cause of readmission.^29^, ^30^, ^31^ The treatment of chemotherapy‐induced neutropenia is, however, complex and may vary considerably across cancer types, aggressiveness of treatment, providers, and institutions. For example, certain providers do not discharge neutropenic patients if they have AML until count recovery. Some patients are discharged home if neutropenia develops during hospitalization but without fever. Patients admitted for fever and neutropenia are hospitalized until count recovery. In other words, the treatment protocol depends on a complex mix of factors. As a result, we decided to pursue a detailed study of the effect of neutropenia on calculable readmission risk prior to discharge from the hospital as a followup investigation that is beyond the scope of this study.

Similar trends observed in the general pediatric population were also observed in pediatric oncology on healthcare utilization risk factors with the exception of previous emergency department (ED) visits. The non‐significant finding on the effect of previous ED visits may indicate that this is one area in which pediatric oncology patients are considerably different from the general population in the risk of readmission. Other variables such as length of stay, previous hospitalizations, and history of an unplanned readmission were all associated with increased risk of readmission. A similar trend was shown for patients who had at least one readmission in the previous 6 months. Of the healthcare utilization factors, the strongest determinant of high risk of readmission was if the index/current visit was a readmission (from a prior discharge), which almost doubled the patient's risk of subsequent readmission. In other words, a patient readmitted to the hospital is at higher risk of a subsequent readmission than their peers.

There were several limitations in this study. One of the limitations is possible bias due to the demographics of the patient population. This implies that the distribution of socioeconomic and racial disparities that impact access to and quality of care may be different from the U.S. population. For example, Black and Hispanic pediatric cancer patients have a significantly higher likelihood of showing late effects from the cancer.^24^, ^25^ The population difference in the database is a result of the location and demographics of the healthcare system contributing data to the database used for the study. The use and reliance on diagnostic coding may not be the most accurate way of capturing patients with cancer, but there were no alternate feasible approaches in the de‐identified EMR database used. An important limitation is that we were not able to capture staging of the cancer in cases of solid tumors, or disease risk/classification/stratification that invariably affects intensity of chemotherapy, disease burden, and presence of comorbidities. We were unable to determine the specific type of tumor targeted by chemotherapy in cases where a patient is diagnosed with more than one type of tumor. Another potential limitation is the time frame of the study. The time frame included data from 2000 to 2017. The prognoses of certain cancer types have changed due to new treatments developed in recent years, including targeted therapy and immunotherapy. Many of the side effects inherent to these new treatment modalities may not have been accurately reported or accounted for. Additionally, certain cancer types had many subtypes that were generalized within the major cancer type, including NHL, whose risk varies by subtype and by the status of the disease (remission or relapse, etc.). We also did not capture specific infection prophylaxis that may have differed between patients and could influence the development of secondary infections.

## 5 CONCLUSION

There are complex relationships between the risk factors for readmission among pediatric oncology patients, including pediatric oncology‐specific variables and other putative risk factors of readmission. This implies the imperativeness and the usefulness of pediatric oncology‐specific readmission models. This pediatric oncology‐specific model for readmission is likely to outperform existing general models and provide more pertinent information from which intervention protocols may be developed. The nested mixed‐effects model provides an excellent starting place for implementation of models to identify patients most in need of interventions in order to prevent future unplanned readmissions.

## CONFLICT OF INTEREST

The authors have stated explicitly that there are no conflicts of interest in connection with this article.

## AUTHOR CONTRIBUTIONS

*Contributed to model development, wrote initial draft, and revised final manuscript*, K.H.; *Served as the oncologist advising on clinical relevance and interpretation of results. She reviewed and revised the draft manuscript*, L.T.; *Contributed to discussions leading to adoption of machine learning algorithm, and interpretation of results. He reviewed the draft manuscript and revised the final manuscript*, W.F.; *Contributed to discussions leading to adoption of machine learning algorithm, and interpretation of results. He reviewed the draft manuscript and revised the final manuscript*, S.T.; *Contributed to discussions leading to adoption of machine learning algorithm, and interpretation of results. He reviewed the draft manuscript and revised the final manuscript*, A.C.; *Contributed to discussions leading to adoption of machine learning algorithm, and interpretation of results. He reviewed the draft manuscript and revised the final manuscript*, M.W.; *Contributed to discussions leading to adoption of machine learning algorithm, and interpretation of results. She reviewed the draft manuscript and revised the final manuscript*, K.P.; *Contributed to discussions leading to adoption of machine learning algorithm, and interpretation of results. She reviewed the draft manuscript and revised the final manuscript*, B.A.; *Conceived of the study, retrieved relevant data, led statistical and machine learning analysis, wrote the initial draft of the manuscript, and revised the final manuscript*, L.E.

## ETHICAL STATEMENT

This study was approved by the Children's Hospital of Orange County's Institutional Review Board with IRB number 180857. Patient consent was waived by the IRB considering the de‐identified nature of the database.

## Data Availability

The data that support the findings of this study are available on reasonable request from the corresponding author, approval from the corresponding author's Institutional Review Board, and approval from Cerner Corporation. The data are not publicly available due to privacy or ethical restrictions.
